# Predictors of colorectal cancer screening in diverse primary care practices

**DOI:** 10.1186/1472-6963-6-116

**Published:** 2006-09-13

**Authors:** Richard K Zimmerman, Mary Patricia Nowalk, Melissa Tabbarah, Seymour Grufferman

**Affiliations:** 1 Department of Family Medicine and Clinical Epidemiology, University of Pittsburgh School of Medicine, 3518 5^th ^Avenue, Pittsburgh, PA, USA; 2 Department Family Medicine, University of New Mexico School of Medicine, Albuquerque, NM, USA; 3 Department of Behavioral and Community Health Sciences, University of Pittsburgh Graduate School of Public Health, Pittsburgh, PA, USA

## Abstract

**Background:**

To explain why rates of colorectal cancer (CRC) screening including fecal occult blood testing (FOBT), flexible sigmoidoscopy (FS), colonoscopy (CS), and barium enema (BE), are low, this study assessed determinants of CRC screening from medical records.

**Methods:**

Data were abstracted from patients aged ≥64 years selected from each clinician from 30 diverse primary care practices (n = 981). Measurements included the rates of annual FOBT, ever receiving FOBT, ever receiving FS/CS/BE under a combination variable, endoscopy/barium enema (EBE).

**Results:**

Over five years, 8% had received annual FOBT, 53% had ever received FOBT and 22% had ever received EBE. Annual FOBT was negatively associated with female gender, odds ratio (OR) = .23; 95% confidence interval = .12–.44 and positively associated with routinely receiving influenza vaccine, OR = 2.55 (1.45–4.47); and more office visits: 3 to <5 visits/year, OR = 2.78 (1.41–5.51), and ≥5 visits/year, OR = 3.35 (1.52-7.42). Ever receiving EBE was negatively associated with age ≥75 years, OR = .66 (.46–.95); being widowed, OR = .59 (.38–.92); and positively associated with more office visits: 3 to <5 visits/year, OR = 1.83 (1.18–2.82) and ≥5 visits/year, OR = 2.01 (1.14–3.55).

**Conclusion:**

Overall CRC screening rates were low, but were related to the number of primary care office visits. FOBT was related to immunization status, suggesting the possible benefit of linking these preventive services.

## Background

Colorectal cancer (CRC) is the second leading cause of cancer-related deaths in the United States, causing an estimated 56,808 annual deaths in 2001 [1]. SEER data show that CRC death rates were 20 per 100,000 in 2001 which is substantially higher than the Healthy People 2010 target of 13.9 deaths per 100,000 [2]. Furthermore, racial disparity occurred with CRC mortality rates at 24.5 per 100,000 for African Americans versus 17.1 per 100,000 for whites.

Healthy People 2010 set screening rate goals for adults aged 50 years and older of 50% for having received a fecal occult blood test (FOBT) within the preceding 2 years and 50% for ever receiving a sigmoidoscopy. Despite these goals and the availability of national recommendations for screening, [3,4] CRC screening rates are low. According to the 2000 National Health Interview Survey, the overall rates of CRC screening among adults age 50 and older in the US were 17.3% for FOBT in the last year, and 30% for lower endoscopy [5].

The objective of this study was to quantify determinants of CRC screening derived from medical records of patients in a variety of geographic, socioeconomic and practice settings, including 30 rural, inner-city, Veterans' Affairs (VA) and urban/suburban primary care practices in western and central Pennsylvania. Demographics, number of office visits, strata, type of visit (acute care, chronic care or preventive) and other factors were evaluated for association with CRC screening, towards the goal of suggesting interventions to raise rates. The perspective of medical record review complements those of patient and provider surveys and focus groups about barriers to CRC screening [6-13].

## Methods

Medical record and survey data from two studies were combined for this study. Methods of sample selection and recruitment were similar for both studies and have been published [14,15]. Thus, they are combined in the descriptions that follow.

### Subjects

A random sample of approximately 22 patients was selected from practices of each of the clinicians in four strata: 8 rural practices in a network, 12 urban/suburban practices from two networks, 7 inner-city practices (3 in a network and 4 independent federally qualified health centers), and 3 VA practices. Requested inclusion criteria were age ≥66 years, to allow for a year of potential access for preventive services that begin at age 65, and an office visit after September 30, 1998. Patients who were homeless, residing in nursing homes, or not currently living in western or central Pennsylvania, and those who were deaf, had severe psychosis or dementia were excluded. This project was approved by the Institutional Review Board of the University of Pittsburgh.

An introductory letter from the principal investigator, along with consent forms and an endorsement letter from the patient's clinician on practice letterhead, was sent to 1800 patients. Potential participants were offered a $20 honorarium to complete an interview and agree to medical record review. The response rates for the surveys were 72%-73% for a total of 1245 [14,15]. Of these, 1002 consented for medical record review (80%), and 981 records were usable with sufficient data for analysis.

### Patient survey

Most results of the patient survey (which dealt with immunizations) have been reported elsewhere and the information from the survey that is reported here relates to demographics and health habits such as smoking [14-16].

### Medical record data collection

Data were collected for all visits (approximately 13,000) dated January 1, 1997 through December 31, 2001. The practices had different methods of recording cancer screening, including handwritten notes, health maintenance flow sheets and electronic medical records. A customized electronic spreadsheet was created for direct data entry using a laptop computer. A code book was developed to guide the medical record reviewers. Trained research assistants collected the following data: stratum, sex, age, presence of a health maintenance flow sheet, date of first visit to practice, and for each visit, date, type, name of clinician seen, use of cancer detection tests, purpose of cancer detection tests (screening or diagnostic), and immunizations given. All visits (excluding laboratory-only visits) during the look-back period (up to 60 months) were recorded, then collated to create a summary data base with total visits, total acute (e.g., viral infection), chronic (e.g., hypertension follow-up) and preventive (e.g., annual physical) visits, number of visits with the study-assigned primary care provider (PCP), demographic variables, cancer detection tests, and immunizations given. Recommendations to screen are reported, but only cancer screenings actually performed were used in the analyses. Rates were adjusted for number of months available for record review.

### Statistical analyses

The original clustered sampling schemes for the studies were no longer applicable to this sample because the data came from two studies and 20% of the sample did not consent to medical record review. Therefore, analyses were conducted without stratification. Trend analysis was performed to determine whether there were differences among the mean number of various types of visits. Frequencies and bivariate analyses using Chi-Square tests or, if the cell size was small, Fisher's exact tests were calculated. Outcome variables were the rate of annual FOBT, ever receiving FOBT, and ever receiving FS, CS, or BE under a combination variable called endoscopy/barium enema (EBE), during the study period.

Multivariate logistic regression was used to determine factors related to CRC screening. In the multivariate models, all variables associated in bivariate analyses with the dependent variable at the *P *≤ 0.10 level were included as independent variables, provided cell sizes were adequate. Also included were any variables specified *a priori *(i.e., age, race). No interaction terms were found to be significant, therefore, they were not included in the models. All statistical analyses were performed using SAS 8.2 statistical software (SAS Inc, Cary, North Carolina). Statistical significance was set at *P *≤ 0.05.

## Results

### Characteristics of the study population

The sample was primarily aged 64–74 years, Caucasian, and lower-income (Table 1). One-half were female, half were currently married and half were previous smokers. Over one-third (35%) had fewer than two visits to the primary care office each year (Table 1); most visits were to the provider designated by the practice as the primary care provider. The average total number of visits per year during the study period was 3.0 ± 2.2, with 1.4 ± 1.9 acute care visits, 8.0 ± 6.1 chronic care visits and 1.0 ± 1.7 preventive visits. Participants had significantly fewer mean acute care visits than chronic and preventive visits (*P *= 0.029) and significantly higher mean chronic care visits than preventive care visits (*P *< 0.001). Although most patients (97%) were seen for chronic care visits, less than half (41%) had preventive care visits and 57% had acute care visits (*P *= 0.03). Over one-half of the women had received a mammogram within the last year and two-thirds had received pneumococcal polysaccharide vaccination (Table 1). Patient medical records indicated that 60% of patients (n = 589) had received one or more types of CRC screening during the study period.

Frequency of FOBT ranged from 0 to 9 during the study period. Only 7.5% (74/981) of patients used FOBT screening at a rate of ≥1 FOBT per year and 53% had ever used FOBT during the study period. Most patients (80.3%) did not have a discussion of FOBT with their providers, with the remainder having ≤ 2.2 discussions per year. Four percent of patients refused FOBT per year. Most (83%) FOBT were undertaken for screening purposes.

Frequency of BE ranged from 0–2, sigmoidoscopy ranged from 0–3 and colonoscopy ranged from 0–4 during the study period. Only 22% (219/981) had EBE recorded within the study period, with 21 (2%) having one or more BEs, 78 (8%) having one or more sigmoidoscopies and 166 (17%) having one or more colonoscopies (individuals may have had more than one type of test). Per year, discussions of EBE were recorded for 24% of patients and 4% of patients refused EBE. Sigmoidoscopy or colonoscopy was primarily performed (68%) for diagnostic purposes.

### Association of colon cancer screening with other factors

In bivariate analyses, receiving annual FOBT was associated with being male, being seen at the VA, never having smoked, receiving annual influenza vaccine and the tetanus toxoid (Table 2). Ever receiving FOBT was associated with being younger, male, married, employed, never having smoked, receiving annual influenza vaccine, having received pneumococcal vaccine and tetanus toxoid, having a moderate number of visits, primarily with one's own provider at an urban/suburban practice. Ever receiving EBE was associated with being younger, male, married, of middle to low income, with fewer visits to the PCP.

Multivariate analyses showed that, after controlling for other variables, a significantly higher rate of annual FOBT was found for those who were male, had more frequent office visits and those who received annual influenza vaccine (Table 3). Ever receiving FOBT was significantly more common in those 64–74 years of age, those who were employed, those receiving care at the VA, those with 3 to <5 visits/year, and in those vaccinated against pneumococcus and tetanus. Multivariate analyses showed that EBE was significantly less common among those 75 years of age and older compared with younger patients; among the widowed, compared with the married; and more common in those with more frequent office visits.

## Discussion

U.S. national rates of CRC screening are low. Based on patient survey data, FOBT within the last year was 19.8% in 1997, 20.6% in 1999 and 23.5% in 2001. In the same three years, lower endoscopy within the last 5 years was reported to be 29.9%, 33.3% and 38.7%, respectively [7]. Although the measurement was different, the present study also found low rates based on primary care office medical record reviews over several years: 7.5% for ≥1 FOBT per year, 53% for ever receiving FOBT and 22% for ever receiving EBE. Interestingly, there was no association between race and CRC screening as has been reported elsewhere [1].

In this study, FOBT was the primary CRC *screening *measure, as 83% of tests were performed for screening purposes. In multivariate analyses, the most important variables associated with FOBT screening were being seen in a VA practice, being employed, having more frequent office visits, annual influenza and other vaccination receipt. EBE in this study was primarily diagnostic in nature, but was associated with being younger, not being widowed and having more frequent office visits. Others have reported an association between the number of visits, particularly preventive care visits, and screening rates. In one study, having a health maintenance visit was strongly predictive of receiving FOBT, flexible sigmoidoscopy, digital rectal exam and PSA [17] and in another, regardless of age, patients who scheduled a preventive visit were more likely to have received preventive services such as mammograms, FOBT and flexible sigmoidoscopy [18].

The Task Force on Community Preventive Services systematically reviewed the literature and recommended a number of interventions to raise rates of CRC screening, including client reminders and removal of structural barriers [19]. Several studies and a meta analysis of interventions to raise CRC screening rates have found that organizational change is highly effective and that provider education; provider reminders, such as health maintenance flow sheets, prevention stickers or stamps, chart reminders; shared responsibility among staff; patient education and reminders, such as patient-held health maintenance cards; and patient financial incentives were also effective [20-27]. Some of the trials to raise rates involved either allowing practices to choose their own interventions from a menu or tailoring them to the office culture and style [26,28].

The VA has a multimodal program for prevention services including assessment, feedback, incentives, reminders, computerized tracking and a prevention nurse who operates under standing orders. Such programs have been highly successful for vaccinations, [29,30] seem to be successful for colon cancer screening as well, as indicated by our data, and are consistent with recent systematic reviews to raise rates.

Given the strength of evidence for interventions to raise rates, the question must be asked why CRC screening rates remain so low. The idea of competing demands suggests that patients and physicians bring an implicit agenda of issues to the primary care visit. Their interaction, complemented by other factors, including visit and health system factors, results in some issues being addressed but not others, which are left for subsequent visits or left unaddressed [31]. Competing demands have been noted for a number of preventive services, [31] in fact, another study found that missed opportunities for vaccination occurred from 38% to 94% of visits, depending on visit type [32].

A barrier to screening may be cost. In the past, patients have reported concerns about payment for CRC screening to be a factor preventing their participation [33]. In July 2001, Medicare reimbursement for screening colonoscopy was approved for persons at average risk for CRC. Therefore, cost may have been a barrier in this population. The importance of cost as a patient barrier to CRC screening should wane. In fact, since these data were collected, Meissner et al have reported significant increases in self-reported colonoscopy rates, especially among adults age 65 and older [34].

Access may also be a barrier to CRC screening using endoscopy. There is currently insufficient capacity to screen all eligible patients using colonoscopy [35] and overall costs would increase, despite reduced CRC care costs [36]. Therefore, FOBT is an important and practical alternative CRC screening method.

Previous research has shown that productivity incentives for providers decrease provision of preventive services such as in-office screenings, [37] because increases in productivity are often associated with decreases in visit time. It follows that physicians need to focus on the most pressing concerns in the time allotted for either an acute or chronic care visit. Longer visit times afford the physician more time to address preventive services such as immunizations, [38] and screenings, suggesting a need for longer scheduled preventive care visits. Mailed reminders to patients to schedule such annual visits might result in increased screening rates.

### Potential to link cancer screening and immunization

The association found between CRC screening and immunizations, suggests that annual influenza vaccination might be a time to encourage cancer screening. There is considerable overlap in ages for influenza vaccine recommendations, CRC screening, and mammography, [3,4,39] and one study found higher rates of mammography when offered to women attending influenza vaccination clinics [40]. For instance, at an influenza vaccination clinic, a second station could be set up to offer FOBT, instructions for completion and mammography prescriptions to appropriate patients, without substantially hindering the vaccination effort. Combining these preventive services would also be more cost efficient given that one mailed reminder could potentially replace two or three. A trial of this idea is warranted.

### Strengths and limitations

Over 13,000 visits from almost 1000 medical records in a diverse cross-section of practice types, socioeconomic and geographic settings were examined. However, racial representation was restricted to primarily two racial groups. This study is limited by the fact that it represents the perspective of medical record review in primary care practices, thereby missing specialist and hospital records of CRC screening that patients may have received, but they were not captured in these data because reports were not forwarded to the PCP. Because cancer screening was relatively uncommon among this population, it was necessary to combine procedures (FS, CS and BE) in order to assess factors related to invasive CRC screening. This prohibited examining factors related to specific screening procedures. While these data were collected several years ago, there has been no significant change in self-reported FOBT rates [34]. Influenza vaccination rate was rather low for several reasons including the fact that 1) many individuals in this geographic area can and do, receive influenza vaccine at community sites rather than at the PCP office; 2) the look-back period included the 2000–01 influenza season in which there was a delay in receipt of vaccine by many PCP offices; and 3) one would expect the rate of receipt of influenza vaccine every year over several years to be lower than rate of receipt during any one year.

## Conclusion

Based on the review of medical records in the primary care office, CRC screening rates were low. The number of visits to the primary care office was associated with both noninvasive and invasive CRC screening tests and immunization status was associated with FOBT screening. This suggests that linking preventive services with similar recommendations, such as annual influenza vaccination and FOBT screening, may improve uptake.

## Competing interests

The author(s) declare that they have no competing interests.

## Authors' contributions

RKZ designed the study, prepared the manuscript, and secured funding for the project.

MPN performed data collection and manuscript editing.

MT conducted data analysis.

SG helped design the study and edited the manuscript.

All authors read and approved the final manuscript.

## Pre-publication history

The pre-publication history for this paper can be accessed here:



## Figures and Tables

**Table 1:** Demographics, Characteristics of Visits to the Primary Care Office, and Preventive Services Usage

**Characteristic**	**N = 981 (%)**
**Demographics and Health Habits from Patient Survey**	
Age	
64–74 years	61
75 + years	39
Race	
Caucasian	92
African American	8
Gender	
Female	51
Marital status	
Never married	5
Married	54
Widowed	33
Divorced/separated	9
Employed	15
Household income	
<$10,000	18
$10,000–19,999	39
$20,000–39,999	29
≥$40,000	14
Stratum	
Rural	30
Suburban	45
Veterans Affairs	20
Inner city	5
Smoking status	
Smoker	11
Quitter	50
Never smoker	39
**Characteristics of Visits to Primary Care Office from Medical Records**	
Visits/year to primary care office^a ^(n)	
< 2	35
2 – 3	26
3 – 4	26
≥5	13
Visits to identified primary care provider^b^	
0 – 25%	8
26 – 50%	12
51 – 75%	19
76 – 100%	62
**Preventive Health Service Usage from Medical Records**	
Annual influenza vaccine (up to 4 seasons)	17
Pneumococcal polysaccharide vaccine	64
Tetanus toxoid	33
Mammogram among women (n = 500)	
None	11
<1 year	59
≥1 year	30

^a^Visits/year was a continuous variable which was grouped as <2, 2-<3, 3-<5 and ≥5, but given simplified labels in the Table.

^b^Visits to identified primary care provider was a continuous variable which was grouped as 0-<26%, 26%-<50%, 50%-<75% and ≥75%, but given simplified labels in the Table.

**Table 2:** Association of Colon Cancer Screening with Demographics, Characteristics of Visits to the Primary Care Office, and Preventive Services Usage from Medical Records

**Characteristic**	**FOBT**^a ^**<1/year ** **(n = 907) %**	**Annual FOBT ** **(n = 74) %**	***P***	**FOBT never ** **(n = 462) %**	**FOBT ever ** **(n = 519) %**	***P***	**No EBE**^b ^ **(n = 762) %**	**EBE ** **(n = 219) %**	***P***
**Demographics**									
Age (ref. = 75 + years)									
64–74 years	61	62	0.852	57	65	0.005	59	69	0.007
Race (ref. = African-American)									
Caucasian	92	90	0.523	93	91	0.24	92	92	0.921
Gender (ref. = male)									
Female	53	23	<.001	60	43	<.001	54	40	<.001
Marital status									
Never married	4	7	0.103	5	4	0.03	4	6	<.001
Married	53	65		51	57		51	65	
Widowed	34	20		37	28		36	22	
Divorced/separated	9	8		7	10		9	7	
Education									
< High school	30	32	0.512	28	32	0.68	32	25	0.06
Some high school	45	50		47	44		45	46	
High school grad/tech school	20	14		20	19		19	21	
Some college	5	4		5	5		4	8	
Stratum									
Rural	19	4	<.001^c^	24	12	<.001	20	12	0.015
Urban	47	23		47	43		43	52	
Veterans' Affairs	19	64		14	30		22	25	
Inner city	15	9		15	14		15	11	
Employed (ref. = unemployed)	15	15	0.931	12	18	0.025	16	14	0.479
Income level									
<$10,000	19	10	0.237	21	17	0.423	20	13	0.02
$10,000–19,999	38	48		39	39		39	38	
$20,000–39,999	29	30		27	31		29	30	
≥$40,000	14	12		13	14		12	19	
Smoking status									
Smoker	11	4	.023^c^	11	11	0.001	11	11	0.326
Quitter	40	32		45	34		41	35	
Never smoker	48	64		44	55		49	54	
**Characteristics of Visits to Primary Care and Preventive Services Usage from Medical Records**									
Visits/year to primary care office^d^									
< 2	36	20	<.001	38	32	0.009	37	29	<.001
2 – 3	27	18		27	24		27	21	
3 – 4	25	38		21	31		24	34	
≥5	12	24		13	12		11	17	
Visits to designated PCP^e,f^									
0 – 25%	8	5	.368^c^	8	8	0.011	7	10	0.608
26 – 50%	12	14		14	10		12	12	
51 – 75%	19	12		15	23		19	20	
76 – 100%	61	69		64	60		62	59	
Mammography status/time since last mammogram									
Never	11	12	0.836	15	5	<.001	11	8	0.072
< 1 year	59	65		54	66		57	70	
≥1 year	30	24		31	29		32	22	
Annual influenza vaccine	16	38	<.001	14	20	0.014	16	21	0.093
Pneumococcal vaccine	63	74	0.093	56	71	<.001	63	69	0.069
Tetanus toxoid within 10 yrs.	32	45	0.025	26	39	<.001	32	36	0.245

^a^Fecal Occult Blood Test

^b^Endoscopy/Barium Enema

^c^By Fisher's Exact Test

^d^Visits/year was a continuous variable which was grouped as <2, 2-<3, 3-<5 and ≥5, but given simplified labels in the Table.

^e^Primary Care Provider

^f^Visits to identified PCP was a continuous variable which was grouped as 0-<26%, 26%-<50%, 50%-<75% and ≥75%, but given simplified labels in the Table.

Totals may differ from 100% due to rounding

**Table 3:** Factors Associated with Colon Cancer Screening by Logistic Regression

**Characteristic**	**Annual FOBT**^**a**^	**FOBT ever**	**Sigmoidoscopy, Colonoscopy or Barium Enema ever**
Age (ref . = 64–74 years)			
75 + years	.99 (.59 – 1.71)	.71 (.53 – .95)*	.66 (.46 – .95)*
Gender (ref. = Male)			
Female	.23 (.12 – .44)*	.74 (.53 – 1.02)	.74 (.49 – 1.11)
Marital status (ref. = Married)			
Never married	1.80 (.64 – 5.07)	.84 (.43 – 1.63)	1.13 (.54 – 2.36)
Widowed	.84 (.42 – 1.68)	.98 (.71 – 1.37)	.59 (.38 – .92)*
Divorced/separated	.99 (.33 – 3.01)	1.02 (.57 – 1.80)	.69 (.33 – 1.44)
Employed (ref. = unemployed)	NS^c^	1.48 (1.00 – 2.20)*	NI^d^
Stratum (ref. = Inner city)			
Rural	N/A^b^	.70 (.41 – 1.18)	1.03 (.5 – 2.1)
Urban		1.18 (.75 – 1.87)	1.51 (.83 – 2.76)
Veterans' Affairs		2.45 (1.45 – 4.13)*	1.28 (.68 – 2.44)
Clinician visits/year (ref. <2)			
2 – <3	1.25 (0.57 – 2.73)	1.17 (0.82 – 1.66)	1.10 (0.69 – 1.74)
3 – <5	2.78 (1.41 – 5.51)*	1.81 (1.23 – 2.60)*	1.83 (1.18 – 2.82)*
≥5	3.35 (1.52 – 7.42)*	1.24 (.77 – 1.98)	2.01 (1.14 – 3.55)*
Annual influenza vaccine	2.55 (1.45 – 4.47)*	1.25 (.85 – 1.84)	1.12 (.72 – 1.74)
Pneumococcal vaccine	1.01 (.56 – 1.82)	1.55 (1.16 – 2.08)*	1.23 (.85 – 1.77)
Tetanus toxoid	1.37 (.81 – 2.33)	1.46 (1.07 – 2.00)*	NS^c^

*P < .05

^a^FOBT = fecal occult blood test

^b^N/A = Not included in logistic regression because of small cell sizes

^c^NS = Not significant in bivariate analyses; not included in logistic regression

^d^NI = Not included in logistic regression because income was correlated with education |r| = 0.44; *P *< .001
